# Change the Laminin, Change the Cardiomyocyte: Improve Untreatable Heart Failure

**DOI:** 10.3390/ijms21176013

**Published:** 2020-08-21

**Authors:** Camila Hochman-Mendez, Ernesto Curty, Doris A. Taylor

**Affiliations:** 1 Regenerative Medicine Research Department, Texas Heart Institute, Houston, TX 77030, USA; ecurty@texasheart.org (E.C.); taylordorisa2020@gmail.com (D.A.T.); 2 RegenMedix Consulting LLC., Houston, TX 77030, USA

**Keywords:** heart failure with preserved ejection fraction, laminin, polylaminin, titin, cardiomyocyte, stiffness, extracellular matrix

## Abstract

No effective medical treatment exists for heart failure with preserved ejection fraction (HFpEF), accounting for approximately half of all heart failure cases. The elevated passive myocardial stiffness in HFpEF is attributed to a combination of alterations in the extracellular matrix (ECM) collagen content and modifications in the sarcomeric protein titin. Here, we propose polylaminin, a biomimetic polymer of laminin, as a promising approach for manipulating the titin isoform shift and phosphorylation in cardiomyocytes. Exploring the pleiotropic effects of polylaminin may be a novel strategy for alleviating symptoms in HFpEF’s multifactorial pathophysiology.

## 1. Introduction

Heart failure (HF) is a final common pathway for many cardiovascular diseases. Around half of the HF population has heart failure with preserved ejection fraction (HFpEF), and its prevalence will likely grow with the increased age and longevity of the population. Recent findings demonstrate that the diastolic dysfunction in HFpEF results from a combination of nitric oxide metabolism dysfunction, systemic inflammation, and diffuse fibrosis [1]. Despite extensive research efforts, the European Society of Cardiology Heart Failure Guidelines [2] stated categorically, “No treatment has yet been be shown, convincingly, to reduce morbidity and mortality in patients with HFpEF”.

The hallmark pathophysiological finding with HFpEF is reduced cardiac compliance and increased diastolic passive stiffness. Total myocardial passive stiffness is the result of extracellular matrix (ECM) stiffness and cardiomyocyte stiffness. Changes in ECM stiffness mostly originate in the interstitial amounts of collagens I and III, highly involved in fibroblast secretion. Cardiomyocyte stiffness, on the other hand, is largely modulated by intracellular titin—a key target for developing therapies for HFpEF and the focus of this perspective. Titin is a large sarcomeric protein that connects the z-lines to myosin in the cardiomyocyte and functions as a molecular spring. Titin’s elastic properties determine the passive mechanical properties of cardiomyocytes [3,4] and can be modulated by two main mechanisms: phosphorylation and isoform shift.

Cardiomyocytes are localized within the basement membrane, a thin, highly specialized layer composed of ECM proteins, in which laminin is a major component [5]. Laminins are cross-shaped molecules that in their biologically active form polymerize into a cell-associated network. Costameres assembled by a combination of integrins and dystroglycans serve as a structural and functional bridge connecting laminins and other proteins to the cell surface. Costameric structures (hence, integrins) participate in signal transduction and transmit force between the contractile apparatus and ECM through interaction with mainly titin and other Z-line associated structures, supporting the mechanical integrity of the sarcolemma and orchestrating mechanical signaling [6], and they also provide spatial cues for muscle fiber organization [7].

**Figure 1:**
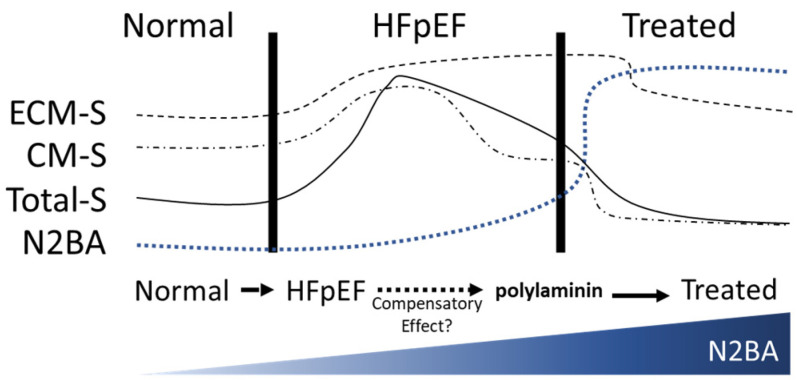
Schematic of the relationship between stiffness and titin-N2BAs during heart failure with preserved ejection fraction (HFpEF) progression, and prediction of its dynamics after treatment with polylaminin. ECM-S, extracellular matrix stiffness; CM-S, cardiomyocyte stiffness; Total-S; total stiffness; N2BA, titin N2BA content.

Because alterations in titin isoform expression can modulate stiffness, we reasoned that manipulating basement membrane proteins, specifically laminin, could alter cardiomyocyte stiffness. To this end, our group’s preliminary in vitro and ex vivo data show that modulating basement membrane laminin isoforms can alter gene expression—titin isoform expression, specifically—in human induced pluripotent stem cell (hiPSC)-derived cardiomyocytes. Therefore, manipulating the laminin content of the ECM may be an effective means of altering the cardiac titin isoform ratio to induce structural and functional changes at the cellular level. In our view, targeting titin isoforms via laminin may be a relevant addition to the HFpEF-management therapeutic arsenal (Figure 1).

## 2. Why Titin Isoforms to Alter Cardiac Compliance?

Phosphorylation and the titin isoform shift, which is regulated by RBM20 [8], are two known mechanisms through which titin-based cardiomyocyte stiffness can be modulated. The phosphorylation of the titin-N2-Bus domain by protein kinase A (PKA) and protein kinase G (PKG) reduces titin stiffness within hours, whereas the PKC phosphorylation of the PEVK regions of titin increases stiffness [9,10]. The isoform shift occurs between titin’s two main isoforms in adults: the longer, more compliant titin-N2BA and the shorter, stiffer titin-N2B. The two isoforms differ mainly in the middle Ig region, which is longer in titin-N2BA and yields a more compliant spring [11,12]. Evidence from the clinical development of HF shows that an increase in titin-N2B increases cardiomyocyte stiffness, whereas an increase in titin-N2BA decreases cardiomyocyte stiffness, thereby increasing cardiomyocyte compliance. The titin N2BA/N2B ratio is 0.3 in healthy adult heart [11]. This ratio increases in patients with HFpEF [10], although the increase in N2BA has been described as a compensatory response to increased ECM stiffness in advanced-stage HFpEF [13]. However, in the case of diffuse global fibrosis commonly observed in patients with HFpEF, many of whom are elderly and comorbid [14], the physiological compensatory titin-N2BA increment appears to be insufficient in counterbalancing the increased ECM stiffness, leading to the diastolic impairment characteristic of the condition.

Shifting the relative titin isoform ratio and altering titin phosphorylation remain promising treatment targets in HFpEF. In patients with HFpEF, titin is phosphorylated at lower levels at protein kinase A sites and at higher levels at protein kinase C (PKC) sites, which is consistent with increased titin stiffness [15]. This rationale has spurred attempts by others to modulate titin stiffness, but the clinical benefit of targeting titin phosphorylation in HFpEF has been, to date, unclear [16]. However, increasing the titin-N2BA/N2B intracellular ratio—beyond the body’s compensatory response—would be expected to alter cardiomyocyte stiffness.

## 3. Polylaminin’s Effects on Cardiac Stiffness

Laminins are critical for the assembly and function of the basement membrane, being tissue- and development-stage-specific. Laminins belong to a family of 16 distinct heterotrimeric proteins. Each laminin isoform comprises an α, β, and γ chain, each of which is named according to numerical subtypes, e.g., laminin-211. During skeletal muscle development, the embryonic laminin isoforms laminin-111 and laminin-511 are progressively replaced with the adult isoform laminin-211 or laminin-221 at the non-synaptic muscle basement membrane [17]. Intriguingly, laminin-α5 has been reported to be upregulated transiently in the basement membrane in human and murine muscular dystrophy, which is accompanied by cardiomyopathy, suggesting a degree of plasticity in basement membrane composition in pathological muscle [18]. Given that both skeletal and cardiac muscle are striated muscle, it is reasonable to extrapolate the role of laminin isoforms from skeletal to cardiac development.

Laminin polymers are the bioactive forms of this glycoprotein. In vitro laminin polymerization was first demonstrated in a cell-free system, dependent on a critical protein concentration, which was optimized by the use of planar lipid bilayers containing sulfated glycolipids and has since been studied extensively in cultured cells [19,20]. More recently, it was demonstrated that laminin could self-polymerize in a cell-free and lipid-free environment forming a polymer called polylaminin that recapitulates the in vivo protein architecture [21,22].

**Figure 2:**
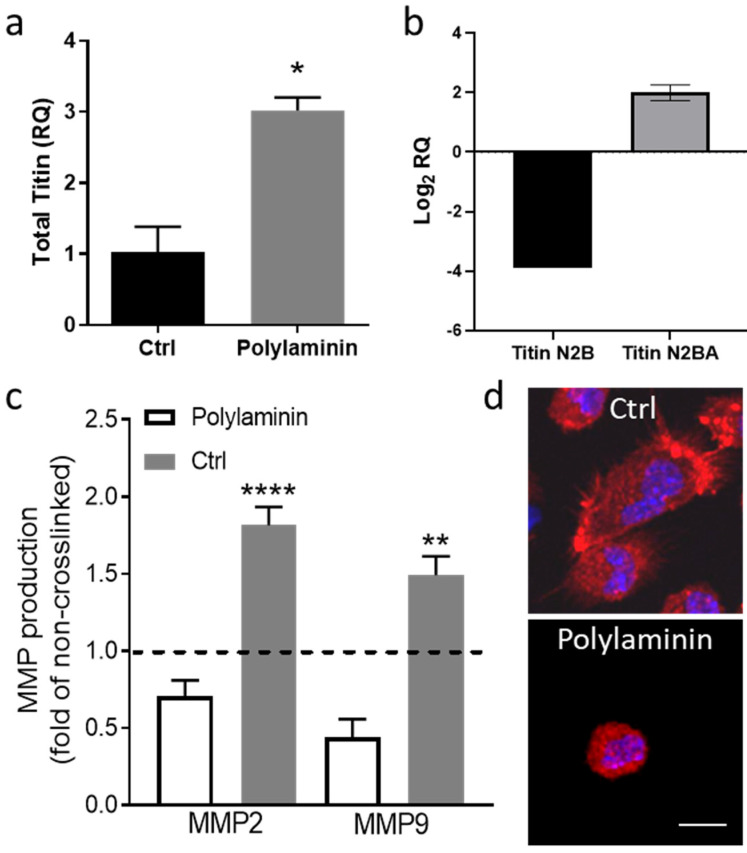
Polylaminin modulates titin expression, and cardiac fibroblast and macrophage phenotypes. (**a**) Gene expression of total titin of cardiomyocytes on gelatin coating (Ctrl) or polylaminin. (**b**) Titin N2B and N2BA gene expression of hearts injected with polylaminin. (**c**) Metalloproteinase 2 (MMP2) and 9 (MMP9) of cardiac fibroblasts on control (Ctrl) or polylaminin. (**d**) Morphology of macrophages on control (Ctrl) or polylaminin. Scale bar = 10 µm. * *p =* 0.0196, ** *p =* 0.021, **** *p* < 0.001.

Preliminary data from our group suggest that polylaminin can modulate the relative expression of titin both in vitro and ex vivo (Figure 2). Cardiomyocytes cultured on polylaminin increased their gene expression of total titin (Figure 2a). The intramyocardial injection of polylaminin in ex vivo rat hearts (Langendorff preparation) induced a 1.9-fold increase in titin N2BA expression and a 3.9-fold decrease in titin N2B expression after 4 h (Figure 2b). Aside from the direct effects on cardiomyocyte stiffness, polylaminin has demonstrated immunomodulatory effects in vivo [23]. Analogously, additional in vitro data from our group supported the idea that polylaminin decreases MMP secretion by cardiac fibroblasts (Figure 2c) and shifts macrophage morphology toward the M2 phenotype (Figure 2d).

## 4. Toward a Multi-Target Therapeutic for HFpEF

Elucidating the mechanisms underlying the regulation of titin by the recognition of patterns in the basement membrane at the costameres will likely open a new window to the mechanisms underlying the regulation of cardiomyocyte stiffness. We propose that exploring the pleiotropic effects of polylaminin after local delivery may be a promising novel therapeutic strategy for alleviating symptoms in HFpEF’s complex pathophysiology. Based on our preliminary findings, polylaminin appears to directly address titin-based cardiomyocyte stiffness. Moreover, polylaminin has anti-inflammatory and anti-fibrotic effects that may be ameliorative for the systemic inflammation and immune dysregulation associated with HFpEF.

## Abbreviations

PKA	Protein kinase A
PKC	Protein kinase C
PKG	Protein kinase G
ECM	Extracellular matrix
HFpEF	Heart failure with preserved ejection fraction
hiPSC	Human induced pluripotent stem cells
